# Development and validation of a nomogram for predicting motoric cognitive risk syndrome among community-dwelling older adults in China: a cross-sectional study

**DOI:** 10.3389/fpubh.2024.1482931

**Published:** 2024-11-27

**Authors:** Huiqi Yuan, Ye Jiang, Yali Li, Lisha Bi, Shuhong Zhu

**Affiliations:** ^1^Health Intelligence Research Center of Beijing Xicheng District, Beijing, China; ^2^Department of Orthopedics, Peking University First Hospital, Beijing, China; ^3^Guangwai Community Health Service Center, Beijing, China

**Keywords:** motoric cognitive risk syndrome, gait, dementia, older adults, logistic regression, nomogram

## Abstract

**Background:**

Motoric cognitive risk (MCR) syndrome is characterized by slow gait speed and subjective cognitive complaints (SCC) and increases the risk of dementia and mortality.

**Objective:**

This study aimed to examine the clinical risk factors and prevalence of MCR in community-dwelling older adults, with the goal of developing and validating a nomogram model for developing prevention strategies against MCR.

**Methods:**

We enrolled community-dwelling participants aged 60–85 years at Guangwai Community Health Service Center between November 2023 and January 2024. A total of 1,315 older adults who met the criteria were randomly divided into a training set (*n* = 920) and a validation set (*n* = 395). By using univariate and stepwise logistic regression analysis in the training set, the MCR nomogram prediction model was developed. The area under the receiver operator characteristic curve (AUC), calibration plots, and Hosmer-Lemeshow goodness of fit test were used to evaluate the nomogram model’s predictive performance, while decision curve analysis (DCA) was used to evaluate the model’s clinical utility.

**Results:**

Education, physical exercise, hyperlipoidemia, osteoarthritis, depression, and Time Up and Go (TUG) test time were identified as independent risk factors and were included to develop a nomogram model. The model exhibited high accuracy with AUC values of 0.909 and 0.908 for the training and validation sets, respectively. Calibration curves confirmed the model’s reliability, and DCA highlighted its clinical utility.

**Conclusion:**

This study constructs a nomogram model for MCR with high predictive accuracy, which provides a reference for large-scale early identification and screening of high-risk groups for MCR.

## 1 Introduction

Due to the aging of the global population, there has been a steady rise in the number of older adults suffering from neurodegenerative diseases such as Alzheimer’s disease and Parkinson’s disease, which emerged as a significant public health issue (1). China has around 25% of the global dementia population, resulting in heavy burdens on families and the healthcare system (2). Currently, there are no clinical treatments that will either cure or prevent the progressive course of dementia, although medication can delay the progression of dementia for some individuals (3). Therefore, it is crucial to focus on early identification of dementia and implement the preventive measures.

Motoric cognitive risk syndrome (MCR) is characterized by slow gait speed and subjective cognitive complaints in individuals without dementia or the absence of daily activity ability (4). Multiple studies have shown that slow gait speed and memory loss are common symptoms seen during the primary phases of dementia (5, 6). A large-scale survey of Japanese community-dwelling older adults revealed that the presence of MCR at baseline significantly increased the risk of developing dementia (7). Meanwhile, slow walking speed and MCR were also linked to a higher risk of death in the medium and long term (8). Therefore, considering MCR as a pre-dementia syndrome in older adults can identify timely older adults with high risk of dementia and implement appropriate intervention efforts to mitigate the rising incidence of dementia.

Numerous current studies have identified several clinic characteristics as risk factors that may predict the MCR risk, such as age, obesity, physical inactivity, hypertension, diabetes, and depression (9, 10). In a multi-center study, poor sleep, hearing, weak grip, and multiple falls were revealed as new connections with MCR (11). There are variations in the risk variables associated with MCR between high-income and middle-or low-income countries. In low-income areas of Malaysia, women residing in rural regions who had obesity, diabetes, heart disease, and cancer were shown to be more susceptible to MCR syndrome (12). In economically developed areas such as Mexico, older age, poor education level, having two or more comorbidities, and diabetes mellitus were related to the high risk of MCR (13).

However, the current literature has primarily concentrated on incidence and risk factors associated with MCR among older adults, merely a few studies have focused on developing a risk prediction model for MCR based on the large-scale sample in China (14). A nomogram is a graphical prediction model that is based on regression analysis and is capable of integrating multiple variables to estimate the probability of an event occurring and visually representing the results (15). Therefore, this study aimed to explore the factors associated associated with MCR and develop a risk prediction model based on a nomogram. This nomogram model will provide valuable evidence for the early identification of MCR syndrome and adopt early intervention and even reduce the incidence of dementia.

## 2 Materials and methods

### 2.1 Study design

The study is an cross-sectional investigation. The reporting of predictive model development and validation was standardized in accordance with Transparent Reporting of a multivariable prediction model for Individual Prognosis Or Diagnosis (TPIPOD) statement (16).

### 2.2 Setting and participants

This study recruited 1,780 community-dwelling older adults via convenience sample from November 2023 to January 2024 in the Guangwai community health service center in Xicheng District, Beijing City, China. The selection of sample size for clinical prediction modeling is typically conducted using the 10 events per variable (10 EPV) method (17). In other words, the required sample size is to ensure at least 10 events for each predictor variable. This study included 34 independent variables, and considering the 20% non-response rate, the minimum required sample size should be 408 cases. Ultimately, we surveyed a total of 1,780 older adults for this study to guarantee the precision of the prediction model and prevent issues like overfitting.

**Figure 1:**
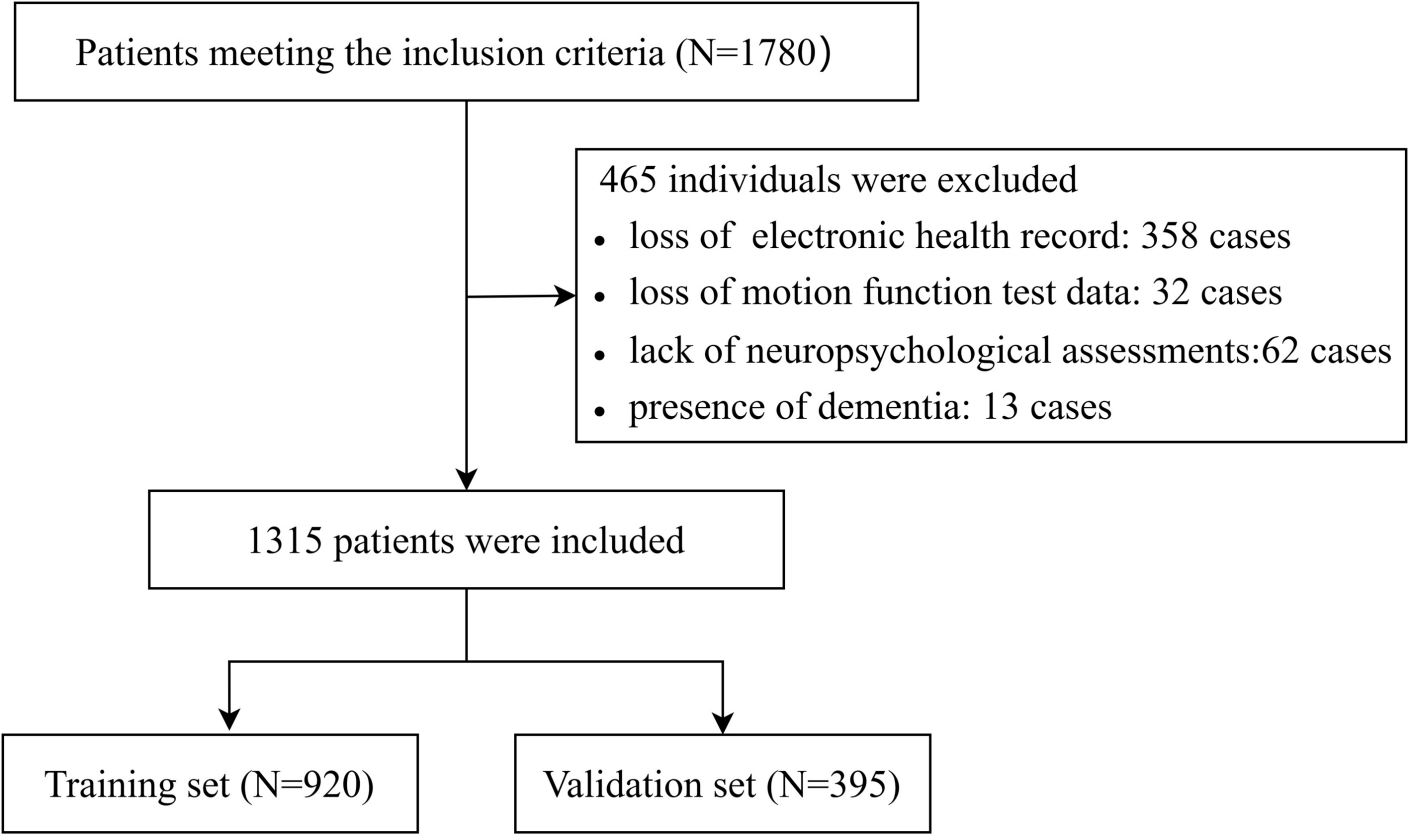
The flowchart of participant selection.

The inclusion criteria were as follows: (1) aged 60–85 years; (2) possessed normal hearing, reading, and writing abilities to complete cognition assessment; and (3) walked without assistive devices (e.g., wheelchairs, crutches). The exclusion criteria were as follows: (1) experienced rapid changes in body function within 30 days (e.g., falls, syncope, or delirium); (2) had dementia, severe cognitive impairment, or mental disorders; (3) the lack of electronic health records, cognitive function assessments, and motor function assessments. As shown in Figure 1, 465 participants were excluded as follows: loss of electronic health record (*n* = 358); lack of assessment of subject cognitive complaints (*n* = 62) or motor function (*n* = 32); diagnosis of dementia using the Mini-Mental State Examination (*n* = 13). We ultimately included and analyzed 1,315 participants.

This study was approved by the ethical review committee of Beijing Rectum Hospital (Beijing Er Long Lu Hospital) before collecting data (2024ELLHA-004-01). All participants provided their written informed consent to participate in this study.

### 2.3 Diagnosis of MCR syndrome

According to the original criteria proposed by Verghese et al. (4), MCR was defined as individuals with subjective cognitive complaints and slow gait speed, but without dementia or mobility disability. In our study, dementia was screened for through a combination of self-report, prior diagnostic history from health records, and the Mini-Mental State Examination (MMSE) (excluding individuals with an MMSE score of ≤17 points in the illiterate group, ≤20 points in the elementary school group, and ≤ 24 points in the junior school and above group) (18). Additionally, subject cognitive complaints were assessed using a self-reported question from the Geriatric Depression Scale-15: “Do you feel that your memory is poorer than that of your peers?” (19). A positive response to this question indicates the presence of subjective cognitive complaints. Gait speed was measured by the average time participants took to walk over a straight 3-meter path three times. Participants were asked to complete the gait test at their normal walking speed. The cutoff slow gait speed was 1.0 standard deviations or below age-and sex-appropriate mean values of gait speed in our study. In this study, the cut-off values for defining slow gait speed for different age groups (60–69, 70–79, and ≥ 80 years old) were 0.857, 0.745, and 0.65 m/s in females and 0.812, 0.749, and 0.621 m/s in males.

### 2.4 Measurement of gait analysis test and the Time Up and Go test

**Figure 2:**
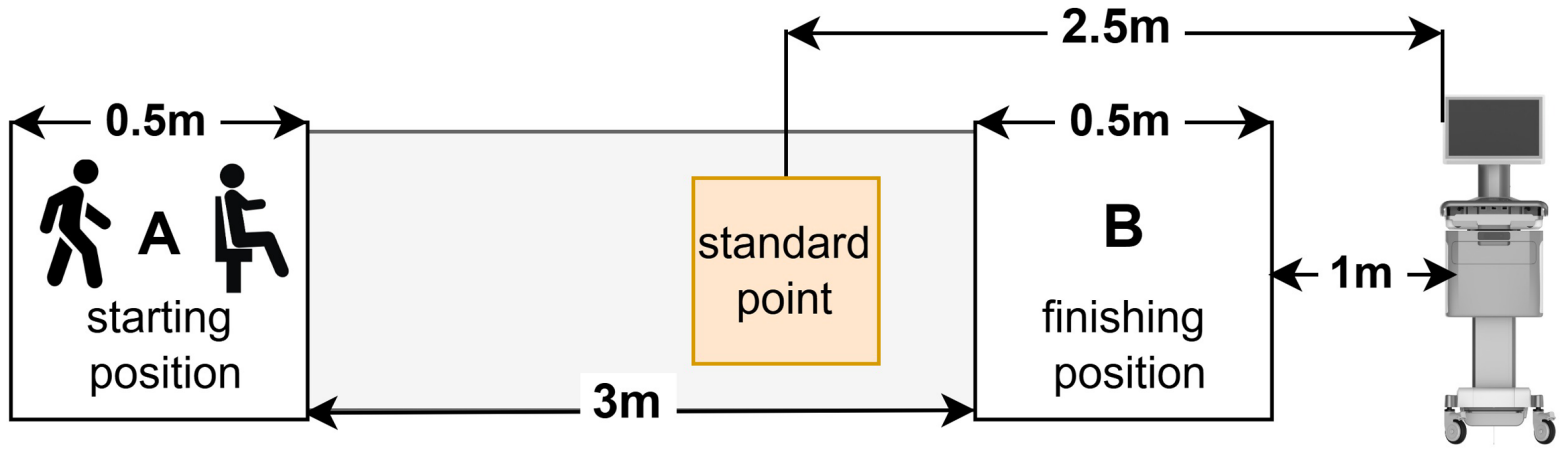
The process of motor function data collection.

In this study, we used the gait analysis test and the Time Up and Go (TUG) test to evaluate motor functions in older adults. Gait parameters and the TUG test were measured using a quantitative evaluation of the motor function system (ReadyGo, Beijing Zhongke Ruiyi Information Technology Co., Ltd.). The ReadyGo system, based on deep visual sensing and motion capture technology, accurately captures individuals’ movement point cloud data, quantitatively assesses human kinematic characteristics and parameters using deep learning algorithms, and automatically generates reports directly. All tests were conducted in a bright indoor environment. After inputting the ID number, age, sex, and education level of the older adults into the equipment, a community doctor explained the process of the gait test and TUG test to the older adults and instructed them to complete it (Figure 2). For the gait analysis test, participants were asked to stand in the starting position A. When the community doctor gave the “start” instruction, they walked at their usual pace on a 3-meter walkway to the finishing position B without using any assistive devices, then turned around and walked back to the starting position A. The test is completed by walking three times without interruption. Upon completion of the test, the equipment autonomously computes the spatiotemporal gait parameters. These included stance phase (%GC), swing phase (%GC), double support phase (%GC), step width (m), step stride (m), step height (m), step cadence (steps/min), gait speed (m/s), stride speed (m/s), swing speed (m/s), turn time (s). For the TUG test, older adults were asked to stand up from a chair with trunk support and armrests in the starting position A, walk on a 3 m straight lane at their usual pace to the finishing position B, then turn around, walk back to the starting position A, and sit down. The whole test process only walked once, and the equipment automatically recorded and generated the TUG test data, which included test time (s), sit-to-stand time (s), stand-to-sit time (s), and turn time (s).

### 2.5 Neuropsychological assessments

Cognitive function was assessed using the Mini-Mental State Examination (MMSE) and the Montreal Cognitive Assessment (MoCA). Community physicians trained by experienced clinical psychologists performed neurological assessments of older adults in a quiet environment. First, a community doctor used the MMSE to screen for dementia (20). Then, MoCA was used to evaluate global cognitive status with a total score ranging from 0 to 30. The MoCA evaluated a variety of cognitive areas, such as visuospatial and executive function, naming, memory, attention, language, abstraction, delayed recall, and orientation, and presented excellent sensitivity and specificity to detect cognitive impairment (21). To account for the influence of education, one point was added to the MoCA scores of participants with 12 years of education or less, and a total MoCA score of less than 26 was considered evidence of mild cognitive impairment (22).

### 2.6 Other measurements

Building on the previous findings (23), demographic characteristics, lifestyle factors, and chronic disease were selected from the residents’ electronic health records and physical examination records as covariates to identify the risk factors in this study. Demographic characteristics included age, gender, education level (elementary school or below/middle/college or above), marital status (married/others), living condition (solitary/non-solitary). Lifestyle factors comprised sleep issues (no/yes), sleep duration (<6 h/≥6 h), smoking status (current smoker or former/non-smoker), drinking frequency (never/occasionally/often), physical exercise frequency (never/occasionally/often), body mass index (BMI; kg/m^2^), and abdominal obesity (no/yes). Sleep issues typically include difficulty sleeping, insomnia, excessive dreams, night wakings, and early wake-up. BMI was calculated as weight in kilograms divided by height in meters squared (kg/m^2^). Abdominal obesity was defined as waist circumference (WC) of >90 cm in men and > 85 cm in women. In addition, previously diagnosed chronic diseases by self-report or the history of the residents’ electronic health records and physical examination records were investigated, including hypertension, diabetes mellitus, hyperlipoidemia, and osteoarthritis disease. Moreover, this study assessed the degree of depression among older adults using the Geriatric Depression Scale (GDS), which has good reliability and validity in screening depression among a large-scale survey of older adults (24). The GDS-15 scored 0–15, with higher scores indicating more severe depressive symptoms. We implemented a cut-off score of >6 to differentiate individuals with depression from those without depression (25).

### 2.7 Statistical methodology

Data analysis was applied using R software (version 3.3.4) and SPSS 25.0. Non-normally distributed continuous variables were expressed as medians with interquartile ranges, and the Mann–Whitney test was employed for inter-group comparison. Categorical variables were reported as number and percentage (%), and inter-group comparison was conducted using χ*^2^* tests or Fisher’s test as appropriate. Subsequently, the variables that showed statistical significance in the univariate analysis in the training group were included in the multivariate logistic regression. Co-linearity among the variables was examined using the variance inflation factors (VIF) with a threshold of 5 (26). We excluded highly associated variables according to VIF before using multivariate logistic regression. The multivariate logistic regression was used to determine independent risk variables, and only those with a statistical significance level of *p*-values <0.05 were eventually chosen. Simultaneously, the 95% confidence interval (CI), odds ratio (OR), and *p*-value of independent risk factors were calculated. The regression coefficients’ results were employed to construct a risk prediction model for MCR, which is represented by a nomogram. The discrimination capability of the model was evaluated using the area under the receiver operating characteristic (ROC) curve (AUC). The Hosmer-Lemeshow goodness-of-fit test and calibration curves were implemented to assess the concordance between predicted and observed probabilities in the nomogram. Additionally, the predictive nomogram’s clinical validity was evaluated using decision curve analysis (DCA).

## 3 Results

### 3.1 Characteristics of study participants

Participant characteristics of the study are presented in Table 1. Overall, the study consisted of 1,315 participants with an average age of 69.59 ± 5.45, and the participation of males (56.5%) in the study was higher than the females (43.5%). Among the 1,315 participants, 123 older adults were diagnosed with MCR. The overall prevalence of MCR syndrome was 9.35%. As shown in Table 1, most of the study characteristics were significantly different (*p* < 0.05) between older adults with and without MCR in the training set, including education, sleep duration, sleep issue, BMI, physical exercises, abdominal obesity status, hyperlipoidemia status, osteoarthritis status, depression status, and MoCA. However, some study characteristics were similar with no statistically significant difference (*p* > 0.05), including age, gender, marital status, living condition, smoking status, drinking frequency, hypertension status, and diabetes status.

**Table 1:** Baseline characteristics of participants.

Variables	Training set (*n* = 920)			Validation set (*n* = 395)		
	Non-MCR (*n* = 834)	MCR (*n* = 86)	*p*	Non-MCR (*n* = 358)	MCR (*n* = 37)	*p*
Age, mean ± SD	69.00 (66.00, 73.00)	69.00 (66.00, 72.00)	0.957	70.00 (66.00, 73.00)	71.00 (68.00, 74.00)	0.070
Gender, *n* (%)			0.626			0.899
Male	462 (55.40)	50 (58.14)		209 (58.38)	22 (59.46)	
Female	372 (44.60)	36 (41.86)		149 (41.62)	15 (40.54)	
Education, *n* (%)			<0.001			<0.001
Elementary or below	40 (4.80)	12 (13.95)		21 (5.87)	8 (21.62)	
Middle^a^	540 (64.75)	70 (81.40)		222 (62.01)	25 (67.57)	
College or above	254 (30.46)	4 (4.65)		115 (32.12)	4 (10.81)	
Marital status, *n* (%)			0.557			0.782
Married	747 (89.57)	75 (87.21)		322 (89.94)	33 (89.19)	
Others^b^	98 (10.43)	11 (12.79)		36 (10.06)	4 (11.8)	
Living condition, *n* (%)			0.548			0.475
Non-solitary	89 (10.67)	11 (12.79)		38 (10.61)	2 (5.41)	
Solitary	745 (89.33)	75 (87.21)		320 (89.39)	35 (94.59)	
Sleep issue, *n* (%)			0.005			0.662
No	453 (54.32)	33 (38.37)		180 (50.28)	20 (54.05)	
Yes	381 (45.68)	53 (61.63)		178 (49.72)	17 (45.95)	
Sleep duration, *n* (%)			<0.001			0.152
<6 h	455 (54.56)	64 (74.42)		198 (55.31)	25 (67.57)	
≥6 h	379 (45.44)	22 (25.58)		160 (44.69)	12 (32.43)	
BMI (kg/m^2^), median (IQR)	25.06 (23.44, 27.03)	26.70 (24.23, 28.22)	0.002	24.80 (23.11, 26.92)	25.10 (24.03, 27.99)	0.199
Smoking, *n* (%)			0.337			0.436
No	653 (78.6)	66 (74.2)		289 (80.73)	28 (75.68)	
Yes	178 (21.4)	23 (25.8)		69 (19.27)	9 (24.32)	
Drinking, *n* (%)			0.346			0.708
Never	628 (75.6)	61 (68.5)		282 (78.77)	29 (78.38)	
Occasionally	125 (15.0)	17 (19.1)		46 (12.85)	5 (13.51)	
Often	78 (9.4)	11 (12.4)		30 (8.38)	3 (8.11)	
Physical exercise, *n* (%)			<0.001			<0.001
Never	84 (10.07)	25 (29.07)		38 (10.61)	10 (27.03)	
Occasionally	272 (32.61)	41 (46.67)		121 (33.80)	18 (48.65)	
Often	478 (57.31)	20 (23.26)		199 (55.59)	9 (24.32)	
Abdominal obesity, *n* (%)			0.001			0.024
No	502 (60.4)	38 (42.7)		223 (62.29)	16 (43.24)	
Yes	329 (39.6)	51 (57.3)		135 (37.71)	21 (56.76)	
Hypertension, *n* (%)			0.113			0.192
No	345 (41.37)	28 (32.56)		156 (43.58)	12 (32.43)	
Yes	489 (58.63)	58 (67.44)		202 (56.42)	25 (67.57)	
Diabetes mellitus, *n* (%)			0.152			0.013
No	586 (70.26)	54 (62.79)		255 (71.23)	19 (51.35)	
Yes	248 (29.74)	32 (37.21)		103 (28.77)	18 (48.65)	
Hyperlipoidemia, *n* (%)			<0.001			<0.001
No	615 (73.74)	39 (45.35)		289 (80.73)	17 (45.95)	
Yes	219 (26.26)	47 (54.65)		69 (19.27)	20 (54.05)	
Osteoarthritis, *n* (%)			<0.001			<0.001
No	805 (96.52)	71 (82.56)		351 (98.04)	29 (78.38)	
Yes	29 (3.48)	15 (17.44)		7 (1.96)	8 (21.62)	
Depression, *n* (%)			0.005			0.086
No	745 (89.33)	68 (79.07)		320 (89.39)	29 (78.38)	
Yes	89 (10.67)	18 (20.93)		38 (10.61)	8 (21.62)	
MoCA, median (IQR)	26.00 (24.00, 28.00)	25.00 (23.00, 27.00)	<0.001	26.00 (25.00, 28.00)	26.00 (24.00, 28.00)	0.165

a Middle: junior school, technical secondary school, high school.

b Others: such us divorced, widowed, or single.

BMI, Body mass index; MoCA, the Montreal Cognitive Assessment.

### 3.2 Comparison of motor function between the MCR and non-MCR groups

As shown in Table 2, during the gait analysis test, significant differences were seen between the MCR and non-MCR groups in all gait variables (*p* < 0.05), including stance phase, swing phase, double support phase, step width, step stride, step height, step cadence, gait speed, stride speed, swing speed, and turn time. During the TUG test, there were significant differences (*p* < 0.05) between the groups for the MCR and non-MCR groups for TUG test time, sit-to-stand time, stand-to-sit time, and turn time.

**Table 2:** Comparison of motor function between the MCR and non-MCR groups.

Variables	Training set (*n* = 920)			Validation set (*n* = 395)		
	Non-MCR (*n* = 834)	MCR (*n* = 86)	*p*	Non-MCR (*n* = 358)	MCR (*n* = 37)	*p*
Gait analysis, median (IQR)						
Stance phase (%GC)	67.47 (66.10, 68.76)	68.97 (67.86, 70.53)	<0.001	67.25 (66.13, 68.52)	69.06 (68.02, 70.01)	<0.001
Swing phase (%GC)	32.52 (31.23, 33.89)	31.02 (29.47, 32.14)	<0.001	32.73 (31.47, 33.86)	30.93 (29.98, 31.98)	<0.001
Double support phase (%GC)	35.14 (32.91, 37.38)	38.19 (36.75, 40.57)	<0.001	35.27 (33.03, 37.53)	38.52 (36.76, 40.01)	<0.001
Step width (m)	0.14 (0.12, 0.15)	0.15 (0.13, 0.16)	<0.001	0.13 (0.12, 0.15)	0.14 (0.13, 0.17)	0.001
Step stride (m)	1.11 (1.01, 1.20)	0.92 (0.80, 0.97)	<0.001	1.11 (1.03, 1.20)	0.89 (0.74, 0.95)	<0.001
Step height (m)	0.12 (0.10, 0.13)	0.10 (0.09, 0.11)	<0.001	0.12 (0.10, 0.13)	0.10 (0.09, 0.11)	<0.001
Step cadence (steps/min)	109.19 (102.94, 116.25)	100.00 (92.85, 104.41)	<0.001	109.19 (102.94, 116.25)	102.94 (95.00, 109.19)	<0.001
Gait speed (m/s)	0.97 (0.87, 1.07)	0.71 (0.64, 0.79)	<0.001	0.99 (0.90, 1.09)	0.69 (0.64, 0.79)	<0.001
Stride speed (m/s)	1.00 (0.91, 1.11)	0.74 (0.66, 0.81)	<0.001	1.01 (0.93, 1.11)	0.74 (0.67, 0.80)	<0.001
Swing speed (m/s)	2.41 (2.19, 2.62)	1.86 (1.68, 2.04)	<0.001	2.45 (2.23, 2.65)	1.86 (1.72, 2.03)	<0.001
Turn time (s)	1.30 (1.10, 1.53)	1.66 (1.43, 2.06)	<0.001	1.30 (1.13, 1.50)	1.66 (1.40, 2.16)	<0.001
TUG test, median (IQR)						
Test time (s)	10.45 (9.21, 11.97)	14.15 (12.44, 15.84)	<0.001	10.37 (9.28, 11.77)	13.86 (12.56, 15.13)	<0.001
Sit-to-stand time (s)	0.56 (0.46, 0.70)	0.66 (0.53, 0.83)	<0.001	0.56 (0.46, 0.70)	0.56 (0.50, 0.73)	0.630
Stand-to-sit time (s)	0.53 (0.46, 0.66)	0.60 (0.51, 0.80)	<0.001	0.53 (0.46, 0.66)	0.60 (0.50, 0.73)	0.071
Turnaround time (s)	1.23 (0.93, 1.56)	1.56 (1.26, 1.95)	<0.001	1.16 (0.90, 1.46)	1.56 (1.36, 2.03)	<0.001

TUG test: Time Up and Go test; %GC: Percent of gait cycle.

### 3.3 Multivariate logistic regression analysis

Taking community-dwelling older adults with MCR syndrome as the dependent variable (assignment: absence = 0, presence = 1), and the statistically significant factors in the univariate analysis as the independent variables, multivariate logistic regression analysis was performed. From the list of candidate variables, 11 gait variables (stance phase, swing phase, double support phase, step width, step stride, step height, step cadence, gait speed, stride speed, swing speed, turn time) were excluded from multivariate logistic regression analysis due to their high multicollinearity (threshold VIF > 5) with outcome variables. As shown in Table 3, multivariate logistic regression analysis showed that education, physical exercise, hyperlipoidemia, osteoarthritis disease, depression, and TUG test time were independent factors for MCR syndrome.

**Table 3:** Multivariable logistic regression analysis of independent risk factors affecting the occurrence of MCR.

Variables	B	SE	*Z*	*p*-value	OR (95%CI)
Intercept	−8.76	0.89	−9.79	<0.001	0.00 (0.00 ~ 0.00)
Education (college and above)					
Elementary or below	1.87	0.55	3.42	<0.001	6.47 (2.22 ~ 18.88)
Middle	2.74	0.68	4.02	<0.001	15.54 (4.09 ~ 59.14)
Physical exercise (never)					
Occasionally	−0.53	0.37	−1.43	0.153	0.59 (0.29 ~ 1.22)
Often	−1.74	0.40	−4.31	<0.001	0.18 (0.08 ~ 0.39)
Hypertension	1.01	0.29	3.53	<0.001	2.74 (1.56 ~ 4.80)
Osteoarthritis	1.26	0.44	2.86	0.004	3.52 (1.49 ~ 8.36)
Depression	1.27	0.36	3.56	<0.001	3.55 (1.77 ~ 7.13)
TUG test time	0.40	0.05	8.10	<0.001	1.50 (1.36 ~ 1.65)

OR, Odds Ratio; CI, Confidence Interval.

### 3.4 Nomogram development and validation

**Figure 3:**
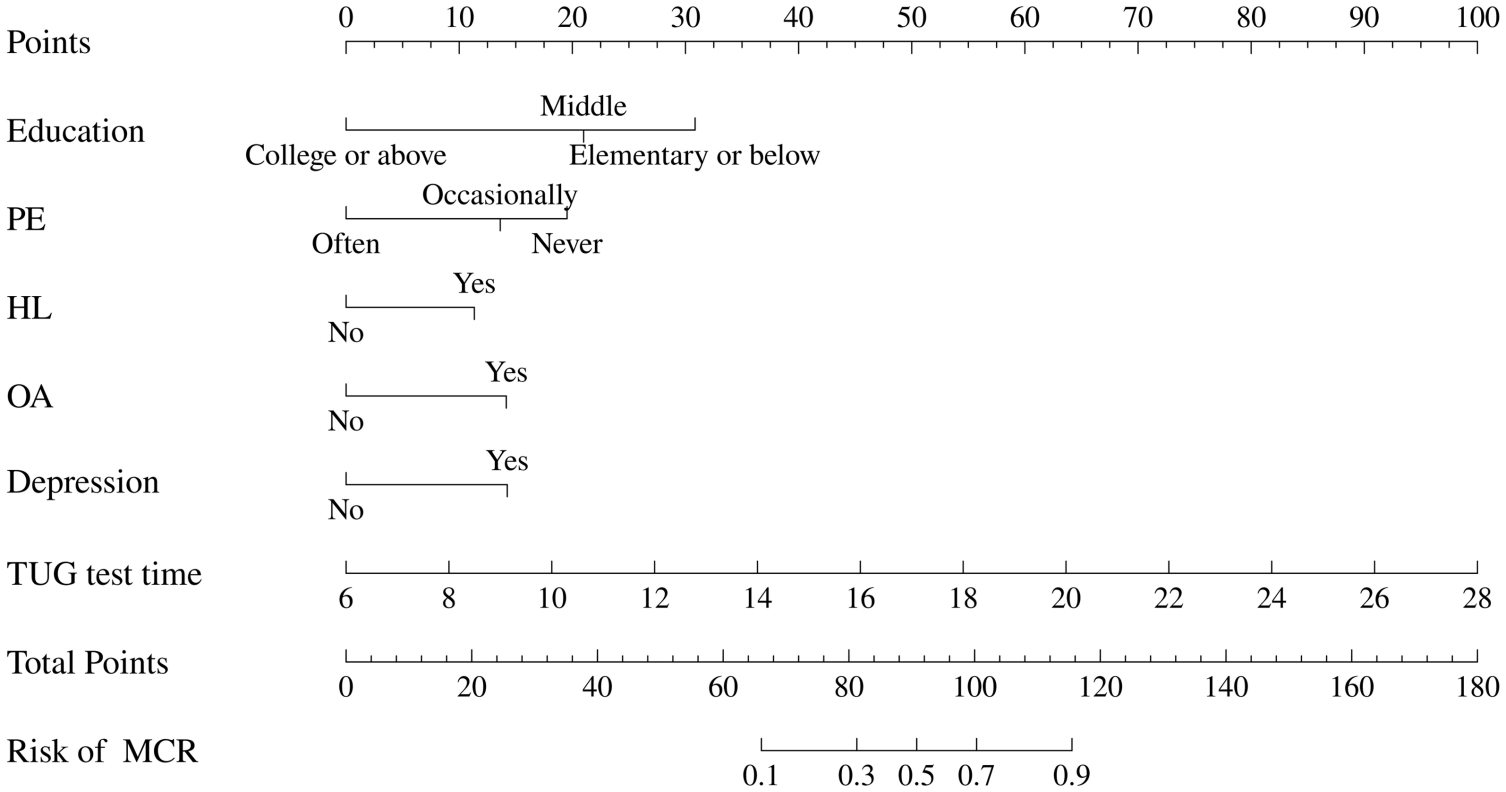
Nomogram for predicting the risk of MCR. The nomogram is employed by identifying the position of each variable on the corresponding axis and drawing a line to the points axis for the number of points. Then, the risk of motoric cognitive risk syndrome at the lower line of the nomogram is calculated by summing the values of all the variables. PE, physical exercise; HL, hyperlipoidemia; OA, osteoarthritis; TUG, Time Up and Go.

Based on the results of multivariable logistic regression, a nomogram was developed to predict the probability of MCR in community-dwelling older adults was constructed, as shown in Figure 3. The predictors included education, physical exercise, hyperlipoidemia, osteoarthritis, depression, and TUG test time. Each factor has a corresponding score, and the cumulative sum of the corresponding scores for all factors was the total score, which correlates with the probability of MCR.

**Figure 4:**
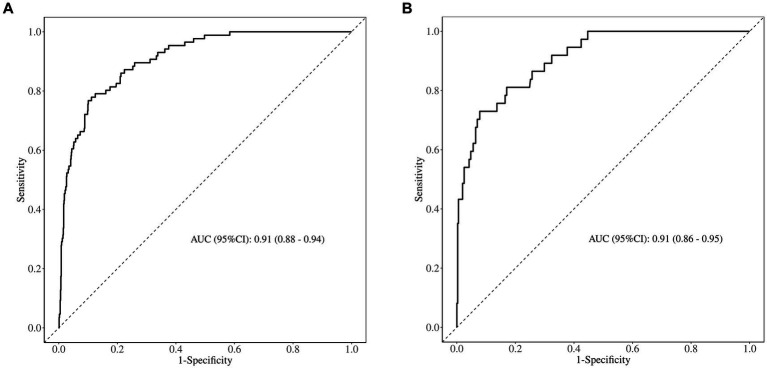
ROC curve of the predictive model for (A) the training set and (B) the validation set. AUC, area under the ROC curve.

**Figure 5:**
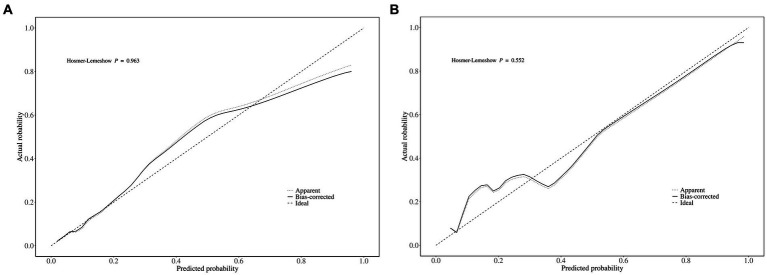
The calibration curves of the MCR nomogram model in (A) the training set and (B) the validation set. Notably, the calibration curve nearly overlays the ideal line, thereby indicating a high consistency between the predicted probabilities and the actual observed incidences of MCR.

The area under the ROC curve (AUC) of the nomogram was 0.909 (95% CI: 0.880–0.939), and 0.908 (95% CI: 0.863–0.953), respectively in the training and validation set, indicating that the model had good discrimination, as shown in Figures 4A,B. The calibration curves align well with the ideal line of the model in both the training set (Figure 5A) and the validation set (Figure 5B), demonstrating that the prediction probability of the model was consistent with the actual probability. Furthermore, the results of the Hosmer-Lemeshow goodness of fit test indicated that good fitting was obtained in both training (χ*^2^* = 2.47, *p* = 0.96) and validation (χ*^2^* = 6.86, *p* = 0.55) set. In the training group, the sensitivity and specificity of the model were 88.8 and 79.9%, positive-predictive value (PPV) was 97.5%, and negative-predictive value (NPV) was 33.5%, indicating that the model had good accuracy. For the validation set, the nomogram showed a sensitivity of 92.2% and a specificity of 70.3%, PPV of 96.8% and an NPV of 48.1%, further evidencing its robustness.

### 3.5 Clinical practice

**Figure 6:**
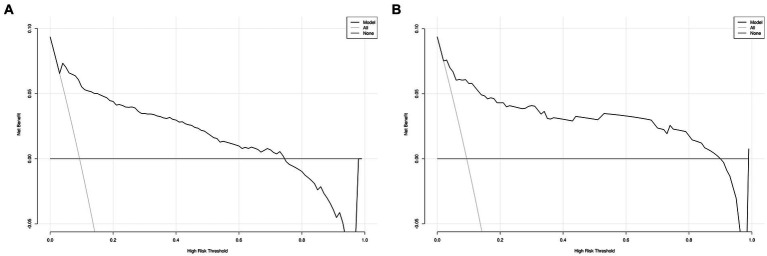
Decision curve for MCR nomogram model in the training (A) the training set and (B) the validation set.

The DCA for the nomogram was conducted to proved clinical usefulness of the nomogram model. As shown in Figure 6A, the net benefit of the training model is higher in the threshold probability interval of 5–75%. Meanwhile, as shown in Figure 6B, the net benefit of the validation model is higher in the threshold probability interval of 5–90%. According to the decision curve, the nomogram model had superior net benefit and predictive accuracy.

## 4 Discussion

In this study, education, physical exercise, hyperlipoidemia, osteoarthritis, depression, and TUG test time were selected as predictor factors to develop a nomogram for predicting the risk of MCR in community-dwelling older adults. Our study showed a MCR syndrome incidence rate of 9.35%, which is lower than the previously reported rate of 12.7% in another Chinese cohort study (27). This might be due to the fact that our study sample was all recruited from communities located in a better-developed city. Most older adults lived a high quality of life with a high level of education, which maintained strong physical and mental capabilities, resulting in a lower prevalence of MCR in our study.

The study revealed that lower educational status was associated with MCR. Various studies have reported that lack of education is related to a higher prevalence of MCR. In a cross-sectional study involving 17,577 participants from Colombian reported that lack of education is related to a higher risk of MCR (28). Similarly, a prospective panel study done in Mexico older adults reported a lower educational status was associated with the presence of MCR (13). Cognitive reserve is recognized as a safeguard against cognitive decline, as it effectively slows down the advancement of neurodegenerative disorders by conserving brain metabolism and enhancing connection in the temporal and frontal regions (29). On the other hand, physical exercise also was associated with MCR. Building on previous relevant studies, the lack of physical activity increased the risk of MCR (9, 29, 30). Regular physical activity can effectively help older adults improve or delay the decline of physical function and mobility and reduce the risk of injury associated with falls (31). What is more, physical activity can enhance angiogenesis and neurogenesis, improve cognitive function, and effectively slow down the progression of neurodegenerative diseases (32–34). Therefore, community family doctors can regularly share exercise videos, providing scientific guidance and supervision to older adults based on their health status and exercise habits. In addition, community health service centers can cooperate with other community organizations to carry out regular group exercise activities, such as morning exercises and square dances, so as to stimulate their enthusiasm for physical exercise.

In addition, this study showed a higher prevalence of MCR among older adults with chronic diseases such as hyperlipidemia, osteoarthritis, and depression. Hyperlipidemia, as a prominent manifestation of the metabolic syndrome, has been identified as a major trigger of cognitive decline and may be affecting overall physical function as well (35, 36). Osteoarthritis, the most common joint disease in older adults, is a significant risk factor for falls and disability (37). Osteoarthritis causes damage and dysfunction of the joint nervous system, affects tissue blood supply, interferes with position sense and pain transmission, and prevents the body from correcting abnormal loads, which in turn leads to joint destruction, so that the brain morphology and physical activity will be altered (38). Our finding aligns with prior research, indicating a strong correlation between depressive symptoms and quantitative gait impairment (19). According to cross-sectional research, MCR was associated with almost 3-fold odds of cognitive impairment in middle-aged adults with depression (39). Additionally, Xu et al. (40) discovered that depression was significantly associated with MCR in both cross-sectional analysis and prospective analysis. Similar to MCI and dementia, several studies have also reported that depression usually occurs in conjunction with mild cognitive impairment, which also accelerates the progression of the spectrum of neurodegenerative diseases (41). Therefore, community health centers can establish health records and classify and manage older adults with chronic diseases through the family doctor team, regular health checkups and record assessments, give reasonable dietary guidance, and develop regular physical exercise programs.

Moreover, this study found that community-dwelling older adults with MCR took longer time to complete the TUG test. The TUG test, as a measure of functional activity with a wide range of clinical utility, has demonstrated excellent reliability and validity in identifying older adults prone to falls (42). Previous studies have also investigated patients with MCR who require more time to complete the TUG test compared to patients with mild cognitive impairment (43). In addition, previous studies have shown that the total time required to complete the TUG test was correlated with cognitive performance, and there was a strong correlation between prefrontal cognitive functioning and changes in TUG subtasks, especially those tasks that require transitions (sit-to-stand, turn-to-walk, and turn-to-sit) (44).

There are numerous advantages to our study. First of all, this study was the first large-scale screening of MCR among community-dwelling older adults in China. Based on the residents’ electronic health records, this study included demographic characteristics, lifestyle factors, and self-reported chronic disease as covariates, which greatly shortens the time of large-scale screening in the community environment. Second, the quantitative evaluation of a motor function system used in our study allowed researchers to obtain more accurate and complex gait data compared to manual assessment. Furthermore, the gait analysis techniques significantly enhanced the efficiency and feasibility of large-scale screening by eliminating the need for wear and calibration. Moreover, nomogram prediction models have been extensively used in clinical research, particularly for prognosticating disease outcomes. Previous studies had mostly emerged to explore the prevalence risk factors of MCR (10, 11, 27, 45), only a few studies have focused on constructing the prediction model of MCR (14). This study developed a low-cost and user-friendly nomogram model of MCR based on six easily obtained variables, with higher accuracy and robust predictive capability than previous research.

Our study also has several limitations. Firstly, this investigation was cross-sectional design, so no causal relationships could be demonstrated. In order to further validate the model, future research should concentrate on longitudinal studies. Second, because this study only included participants from a single community health center, there may be selection bias, which could limit the applicability of the findings to other settings or populations. Future research could continue to recruit more older adults or cooperate with other centers to bolster the reliability and generalizability of the results. Finally, this study relied solely on self-reported subjective cognitive complaints that introduce a potential for recall bias and subjective interpretation, impacting the reliability of MCR diagnosis. Hence, it is warranted that objective cognitive complaint assessments be incorporated into future research in order to substantially enhance the study.

## 5 Conclusion

This study constructed a nomogram model for predicting the MCR risk of the older adults in the community by using six critical preoperative predictors. The ROC curve, calibration curve, and goodness-of-fit test results, which have been verified in both the training and validation databases, demonstrate that it has both good prediction ability and accuracy. Furthermore, the DCA curve demonstrates that it is clinically feasible and can be employed as a valuable instrument for the early detection and intervention of MCR in older adults, particularly in primary healthcare institutions.

## Data Availability

The raw data supporting the conclusions of this article will be made available by the corresponding author, without undue reservation.
